# A Non-Invasive Analysis of Seed Vigor by Infrared Thermography

**DOI:** 10.3390/plants9060768

**Published:** 2020-06-19

**Authors:** Liya Liu, Zhongsi Wang, Jing Li, Xi Zhang, Ruohan Wang

**Affiliations:** National Engineering Laboratory for Tree Breeding, Key Laboratory for Genetics and Breeding of Forest Trees and Ornamental Plants, Ministry of Education, College of Biological Sciences and Biotechnology, Beijing Forestry University, Beijing 100083, China; 18763820935@163.com (L.L.); wang_zsi@bjfu.edu.cn (Z.W.); xsp0901@bjfu.edu.cn (J.L.); zhangxi@bjfu.edu.cn (X.Z.)

**Keywords:** infrared thermal imaging, non-invasive seed screening, seed vigor, thermal decay, *Ulmus pumila* L., *Oryza sativa* L.

## Abstract

This paper establishes robust regression models for fast and efficient estimation of seed vigor based on high-resolution infrared thermography. High seed quality is of great significance for agricultural and silvicultural purposes, and seed vigor is a crucial agent of seed quality. In this study, we used the non-invasive technology of infrared thermal imaging to analyze seed vigor of *Ulmus pumila* L. and *Oryza sativa* L. Temperatures of young age and aged seeds during thermal decay were monitored over time. We found that the thermal decay dynamics of *U. pumila* seeds were highly differential among seeds with differential vigor. Furthermore, a regression model was developed to estimate seed vigor based on its thermal decay dynamics. Similarly, a close relationship was also found between thermal decay processes and seed vigor in *O. sativa*. These results suggest that infrared thermography can be widely applied in non-invasive examination of seed vigor and allows fast and efficient seed screening for agricultural and silvicultural purposes in the future.

## 1. Introduction

Intense competition in the field of agricultural technology has promoted the global development of seed-related industries, which has consequently given rise to an urgent need for improved seed quality. High-quality seeds are the building blocks of good seedling performance. One of the most important agents of seed quality is seed vigor, which profoundly influences the potential for tree growth and crop production [1]. Previous studies have demonstrated that seed vigor gradually decreases after harvest and storage [2,3]. Seeds with low vigor always exhibit low germinability, which directly affects agricultural and forestry yields [3]. In *Zea mays*, a 25% decrease in seed vigor (as indicated by electrical conductivity) resulted in a 37% decline in initial growth [4]. Based on these demonstrated effects of low seed vigor, seed scientists worldwide are focusing on research to improve seed vigor.

Currently, two main approaches are taken to improve seed vigor: genetic improvement and seed screening [5]. Owing to the rapid development of molecular biology and bioinformatics techniques, genetic improvement methods have made substantial progress in recent years [6,7]. Many genes affecting seed development have been characterized and isolated during the past decades [7,8], and even single or multiple beneficial genes can be introduced into crops via modern genetic technologies [9,10]. However, one persistent challenge is that the improvement of a desirable trait via genetic improvement is always accompanied by the impairment of other beneficial traits [11]. Moreover, these methods require considerable time and investment for commercially available quality-improved seeds. Thus, seed screening provides an alternative for obtaining high-quality seeds at low costs of time and investment.

Several methods have been developed for seed screening by evaluating seed vigor. However, traditional methods, such as tests of conductivity [12] and staining with triphenyl tetrazolium chloride (TTC) [13] or bromothymol blue reagent [14], do not meet the demands of modern agriculture or silviculture, for which efficient and non-invasive seed vigor identification is greatly needed [15,16]. Several new technologies are currently emerging, all of which can potentially be applied in non-invasive seed vigor tests [17,18,19], including infrared thermal imaging technology [20]. Early studies have demonstrated that heat can be produced in seeds during metabolic activities, which show notable dynamics in response to the changing physiological status of seeds [21]. Heat emission in plant tissues can be monitored by thermal imaging technologies for several agricultural purposes, e.g., detection of plant responses to salt and drought stresses [22], plant diseases [23] and development [24,25], and monitoring of crop growth in farmland [26]. Recently, we developed an infrared thermal imaging platform for high-resolution detection of heat emission in plant tissues, at a spatial resolution of 47 μm [27]. The development of this thermal imaging technology enables rapid non-invasive tests of seed vigor in modern seed screening.

Infrared rays are 0.75–1000 μm wavelength invisible rays, including near infrared (0.75–1.5 μm), middle infrared (1.5–6 μm), and far infrared (6–1000 μm) rays [27]. Any object having a temperature higher than absolute zero (0 K or −273.15 °C or −459 °F) emits radiation in the infrared range of the electromagnetic spectrum, which provides a characteristic spectrum containing identification information on the object [28]. Thus, the thermal status of objects can be measured by detecting and analyzing its emitted infrared spectrum [29]. In the current study, we adopted a high-resolution infrared thermography system to monitor the thermal dynamics of young age and aging seeds of *U. pumila* after exposure to halogen pulses. By relating the thermal profiles to seed vigor, we established a non-invasive method for robust and rapid seed screening. Furthermore, we applied this method to rice (*Oryza sativa*) seeds and found that it can discriminate seeds with various vigor.

## 2. Materials and Methods

### 2.1. Seed Materials

*U. pumila* seeds used in this study were collected from 30-year-old trees on the campus of Beijing Forestry University (39°59’ N, 116°20’ E). Seeds were air dried at room temperature for four days and then cleaned by removing misshaped and empty seeds. The cleaned seeds were dry-stored in nylon bags at −20 °C until use. *Cunninghamia lanceolata* (Lamb.) Hook. *Robinia pseudoacacia* L., *Glycine max* (Linn.) Merr., *Oryza sativa* L., *Zea Mays* L., and *Lycopersicon esculentum* Mill. seeds were stored at 4 °C for approximately 1 year before use.

### 2.2. Seed Germination and Vigor Tests

Germination and vigor tests were conducted for *U. pumila* seeds after 0, 24, 48, 72, 96, and 120 h of aging. The seeds were first placed on moistened filter paper in Petri dishes for 6 h to allow full imbibition (Figure S1). They were then allocated to ageing treatment in a warm bath at 45 °C for various periods. During the aging treatment, seeds were kept in the same Petri dishes as for imbibition, which were placed in the bath. Three replicates of 50 seeds were used for each of the germination and vigor tests. For germination tests, the seeds were first placed on moistened filter paper in Petri dishes for 6 h for full imbibition, and then they were incubated in water at 26 °C under an 8 h/16 h light/dark photoperiod (approx. 100 µmol m^−2^ s^−1^ cool white fluorescent light); germination percentages were recorded after two days. For vigor tests, seed coats were peeled off after imbibition and the seed were immersed in 2% TTC staining solution and kept at 35 °C in darkness for 30 min. TTC staining is usually used as a redox indicator of active tissues. TTC-stained seeds were rinsed with distilled water three times, and red-stained seeds were counted.

### 2.3. Setup of the Thermal Imaging System

**Figure 1:**
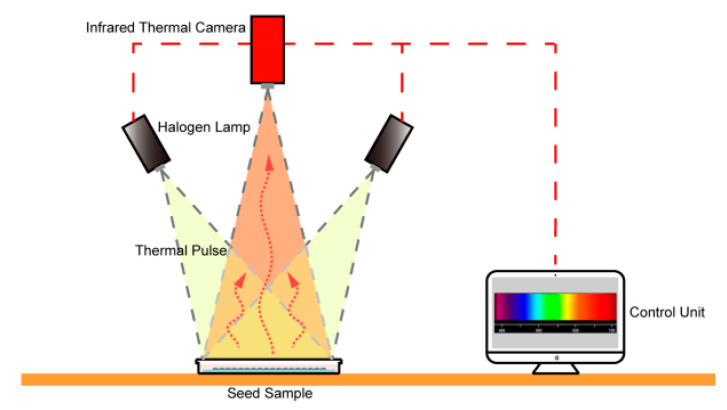
Schematic diagram of the customized infrared thermography device. Seeds were exposed to pulse irradiation from halogen lamps (black rectangle) for 5 s to achieve thermal stimulation. The lamps were controlled by the same switch, and they were removed when turned off. Stimulated thermal signals (infrared light) from the seeds were collected by the thermal camera and stored in the control unit.

An infrared thermography imager (TiX660, Fluke, Everett, Washington, USA) with a detection spectrum of 7.5–14 μm was used to set up the thermal imaging system. A working scheme of the thermography system for real-time monitoring of seed temperature dynamics is shown in Figure 1. Briefly, two halogen lamps (50 W each; Voltage = 220 V) were used to irradiate seeds from a distance of 0.2 m at an angle of 45 degrees. The lamps were connected with an integrated voltage stabilizer (± 0.5%) and controlled by the same switch, and they were removed when turned off. The irradiation pulse was set at 15 Hz. This type of thermal stimulus does not cause damage to seeds [30]. The thermography imager from a distance of 0.5 m, above the sample area was able to take real-time images at a frame rate of 15 Hz and thermal sensitivity of 0.01 °C.

### 2.4. Thermal Imaging of Seeds

Using the customized thermal imaging system, real-time infrared images were taken of young age seeds (0 h) and aging *U. pumila* seeds (24, 72, 96, and 120 h of aging). All the seeds were blotted dry on filter paper before thermal imaging. Seed temperature was homogenized before thermal imaging by placing the seeds at 28 °C for 30 min. The seeds were placed on dry filter paper (Fuyangbei Wood Pulb Co., Hangzhou, China) and exposed to irradiation pulses from halogen lamps for five seconds prior to thermal imaging, and the lamps were removed when turned off. Thermal images were taken at 15 Hz for 90 s and a total of 1350 thermal images were obtained in a time sequence for each experiment, which contained nine seeds in the field of view. For each seed, a circular area of interest was sampled (d = 1 mm) in the major heat-emitting portion at 0 s, and the temperatures of all pixels (including 300 pixels) in this area were averaged to represent the seed temperature. During the thermal decay process, temperatures were extracted in the same sample area for the seeds.

### 2.5. Influence of Water Content on Seed Vigor Estimation

Thermal images were compared between dry and fully (6 h) imbibed *U. pumila* seeds. Seed temperature was homogenized before thermal imaging by placing the seeds at 28 °C for 30 min. The seeds were first exposed to irradiation pulses from halogen lamps for 5 s before thermal imaging. Thermal images were taken every 5 s for 60 s, nine seeds were used for each of the dry and imbibed seeds. Temperature data were extracted as described above.

### 2.6. Validation of the Method Using O. sativa Seeds

Seeds of *O. sativa* were used to validate our method of seed vigor estimation using far-infrared thermography. The seeds were artificially aged in the same way as *U. pumila* seeds, except that seeds were aged for 0, 3, 6, 12, 18, and 24 h to obtain seeds with various vigor. Germination assays, vigor tests, and thermal imaging were conducted in the same way as described for *U. pumila* seeds. When extracting real-time temperatures to characterize thermal decay processes of the seeds.

### 2.7. Thermal Imaging of Other Seeds

Thermal imaging was performed on other seeds that were not exposed to halogen lamp pulses to measure their temperatures. Seeds of *C. lanceolata*, *R. pseudoacacia*, *G. max*, *O. sativa*, *Z. mays* (wild type and *legumin 1* mutant), and *L. esculentum* wild type and *sabp 2* mutant were used. One thermal image was taken for each species or genotype.

### 2.8. Data Analysis

Temperature data were extracted from thermal images using Smart view (v3.14, Fluke). Data normalization was performed as *T_n_* = (*T_i_* − *T*_min_)/(*T*_max_ − *T*_min_), where *T_n_* is the normalized temperature at time *n*, *T_i_* is the real-time temperature at time *i*, *T*_max_ is the maximum temperature, and *T*_min_ is the minimum temperature of a seed during the thermal decay process. R-Studio (v7.2 Build 153957) was used to fit temperature dynamics during thermal decay for all seeds using the least squares method. The ranges and coefficient of variation (CV) of *T*_max_ and *T*_min_ are shown in Table S1. A general equation (Equation (1)) describing time-dependent thermal decay was adopted from [31], where *a* and *c* are the first and second decay amplitudes of thermal decay, respectively; *b* and *d* are the first and second decay lifetimes of thermal decay, respectively; and *t* is the time on the decay curve. SPSS 18.0 (SPSS Inc., Chicago, IL, USA) was applied to analyze the variation in the regression coefficients and correlation between regression coefficients and seed germination rates. Equation (1):

(1) $$y=a\cdot \exp\left(\frac{-t}{b}\right)+c\cdot \exp\left(\frac{-t}{d}\right)$$

## 3. Results

### 3.1. Germination and Vigor of U. pumila Seeds Decreased After Aging

**Figure 2:**
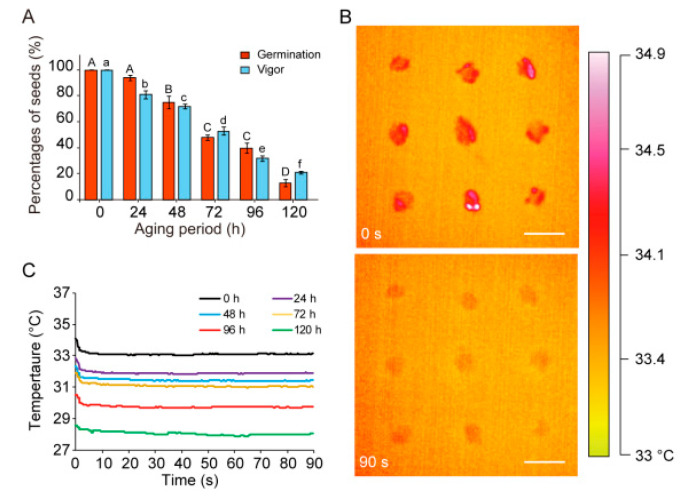
Real-time monitoring of young age and aging *U. pumila* seed temperatures using the customized thermography system. Germination percentages and vigor decreased after aging; data are means ± SE (**A**). Different uppercase letters indicate significant differences (*p* < 0.05) in germination percentages across aging treatment times and different lowercase letters indicate significant differences (*p* < 0.05) in vigor. Representative thermal images of young age *U. pumila* seeds at 0 and 90 s during thermal decay (**B**). Temperatures were color coded, with red indicating high temperature. Young age and aged seeds exhibited different temperatures after 5 s of pulse irradiation exposure (**C**). Real-time temperature dynamics of seeds (n = 12) after pulse irradiation (**C**). Bar = 0.5 cm.

The germination percentages of *U. pumila* seeds significantly differed (*p* < 0.05) among the aging treatment periods. Young age seeds germinated to 100% after two days (Figure 2A). The seeds maintained a high germination percentage, as high as 93.67 ± 2.08%, after 24 h of aging. However, after longer aging periods, germination percentage decreased gradually, to a minimum of 12.67 ± 3.06% after 120 h (Figure 2A).

In the TTC staining experiment, 100% of healthy *U. pumila* seeds were dyed red, suggesting high seed vigor before aging (Figure 2A). During aging, the proportion of seeds that were stained red decreased considerably. When seeds were exposed to aging treatment conditions for 120 h, viability decreased to 20.00 ± 2.00%, indicating a remarkable decrease in seed vigor during aging (Figure 2A).

### 3.2. Thermal Imaging of U. Pumila Seeds

The customized thermal imaging system profiles seed temperatures at a spatial resolution of 47 μm with a field of view of 46 cm^2^ (7.87 cm × 5.90 cm), which provides high-resolution temperature measurements for seed lots (Figure 2B). The temperature of young age *U. pumila* seeds was 34.05 ± 0.19 °C (n = 12) after 5 s of irradiation by halogen lamps and decreased significantly (*p* < 0.05) to 33.21 ± 0.13 °C after 90 s of thermal decay. After the aging treatment, seeds showed relatively lower temperatures in response to halogen lamp irradiation compared with young age seeds, decreasing to 28.56 ± 0.96 °C after 120 h of aging (Figure 2C). Using real-time thermal imaging, we found that seed temperature showed time-dependent decay after halogen pulse irradiation (Figure 2C).

### 3.3. Time-Dependent Thermal Decay of U. Pumila Seeds

**Figure 3:**
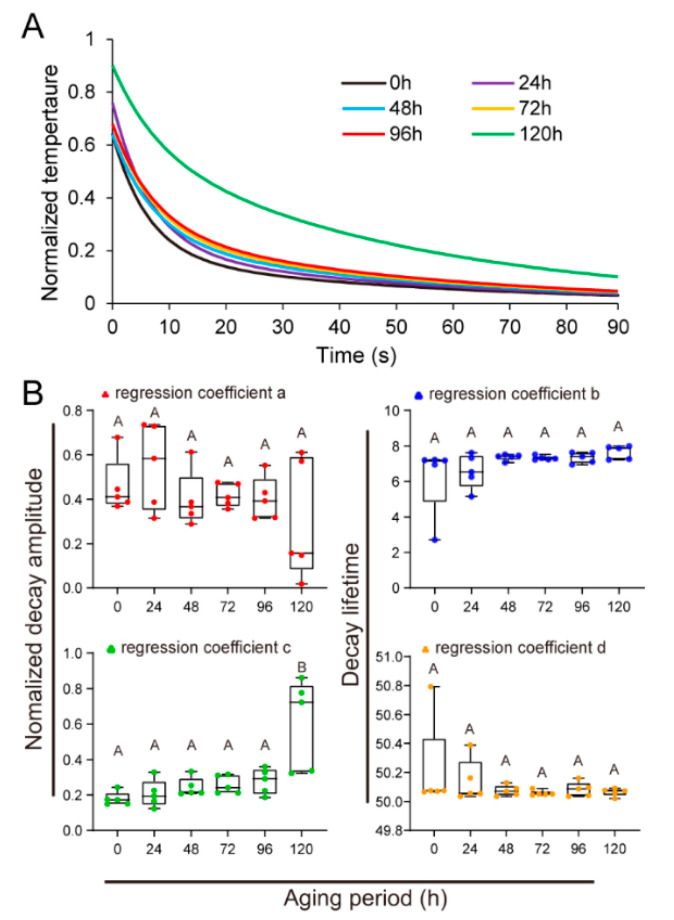
Quantitative description of thermal decay processes in *U. pumila* seeds. Young age and aged seeds exhibited different thermal decay patterns as indicated by the normalized temperatures (**A**). Time-dependent thermal decay processes in seeds (n = 8) were quantitatively described by fitting normalized real-time temperatures to the empirical equation (**B**). Thermal decay parameters (*a* and *c* are the first and second decay amplitudes, respectively (left), *b* and *d* are the first and second decay lifetimes, respectively (right)) varied significantly (*p* < 0.05) among seeds during aging. Data are means ± SE, and different uppercase letters on the bar graph indicate significant differences (*p* < 0.05) in (**B**).

By normalizing the real-time temperature data, we found that young age seeds and aging seeds of *U. pumila* exhibit different time-dependent thermal decay patterns (Figure 3A). The thermal decay processes were quantified by fitting normalized real-time temperatures to the empirical equation (Equation (1)). The first thermal decay amplitude *a* in the equation was 0.46 ± 0.06 for young age seeds, and values decreased during the aging process to 0.30 ± 0.12 after 120 h aging (Figure 3B). In contrast, the second decay amplitude *c* increased during the aging process from 0.18 ± 0.02 in young age seeds to 0.60 ± 0.11 in seeds aged for 120 h. Spearman correlation analyses revealed significant (*p* < 0.05) correlations between these parameters and seed vigor, with the second decay lifetime *c* exhibiting the strongest relationship (*p* < 0.01, Table 1).

### 3.4. Influence of Water Content on Seed Vigor Estimation

**Figure 4:**
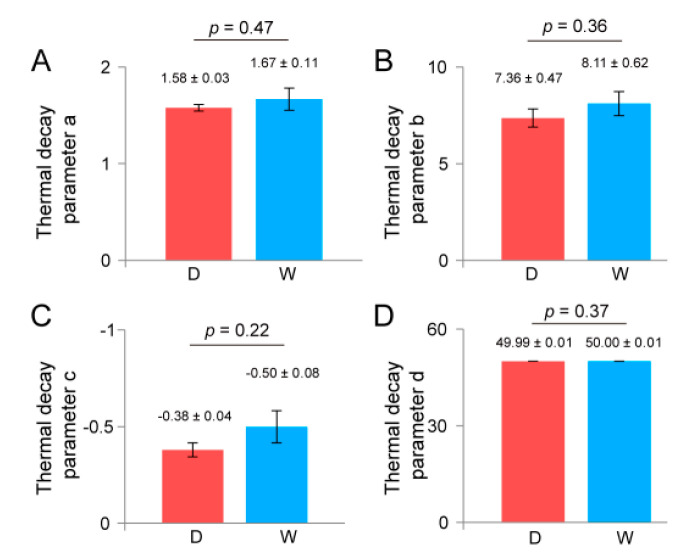
Comparison of thermal decay parameters between dry (D) and wet (W) *U. pumila* seeds. (**A**). Histogram differences of thermal decay parameters *a*. (**B**). Histogram differences of thermal decay parameters *b*. (**C**). Histogram differences of thermal decay parameters *c*. (**D**). Histogram differences of thermal decay parameters *d*. Data are means ± SE, and digital indicate significant differences (*p* < 0.05).

Considering that water content would influence the emission of infrared light in seeds, we estimated whether our method could correctly determine seed vigor when the seeds are differentially imbibed. Using data normalization and regression analyses, we obtained thermal decay equations for both dry and fully imbibed *U. pumila* seeds. The thermal decay parameters of *a*, *b*, *c*, and *d* did not show significant differences (*p* > 0.05) between dry and imbibed seeds (Figure 4). Especially the parameter *c* remained the most constant, with a value of −0.38 ± 0.04 for dry seeds and −0.50 ± 0.08 for fully imbibed seeds.

### 3.5. Application of Seed Vigor Test by Infrared Thermography in O. sativa Seeds

**Figure 5:**
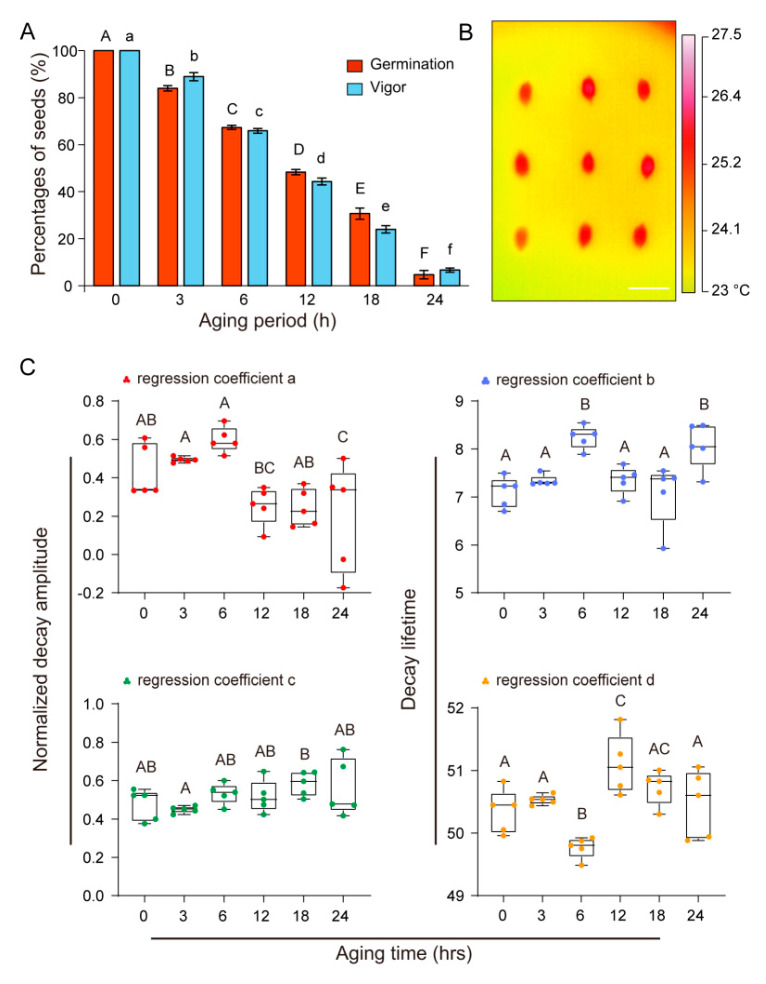
Characterization of thermal decay processes of *O. sativa* seeds using the customized thermography system and germination percentages, where vigor decreased after aging. Seed germination and vigor decreased gradually during 24-h artificial aging treatment (**A**). A thermal image of young age seeds after exposure to halogen lamp irradiation. The capital and lowercase letters on the bar graph represent the difference in germination and vigor, respectively (**B**). Variation in thermal decay parameters for rice seeds with various vigor (**C**). Thermal decay parameters (*a* and *c* are the first and second decay amplitudes, respectively (left), *b* and *d* are the first and second decay lifetimes (right), respectively) varied significantly (*p* < 0.05) among seeds during aging. Data are means ± SE, and different uppercase letter on the bar graph indicate significant differences (*p* < 0.05) in (**C**).

We further validated our method by vigor tests of *O. sativa*. A 24-h aging treatment decreased seed vigor from 100% to 6.67 ± 0.88% for *O. sativa*, which effectively provided seeds with various vigor for the tests (Figure 5A). Seed temperatures of *O. sativa* were clearly captured during thermal decay processes using our thermal imaging system (Figure 5B). There were also considerable changes in the thermal decay parameters among *O. sativa* seeds with various vigor (Figure 5C). The thermal decay parameter c showed the closest correlation with seed vigor (Table 2).

The thermal decay parameter *c* showed close correlation with seed vigor in both *U. pumila* and *O. sativa* seeds analyzed in this study, which promoted us to search for quantitative estimation of seed vigor via the parameter *c*. Regression analyses were performed between the parameter *c* and seed vigor using various models (Table 3). The polynomial function model showed the best fit in *U. pumila* and *O. sativa*. (Table 3).

**Figure 6:**
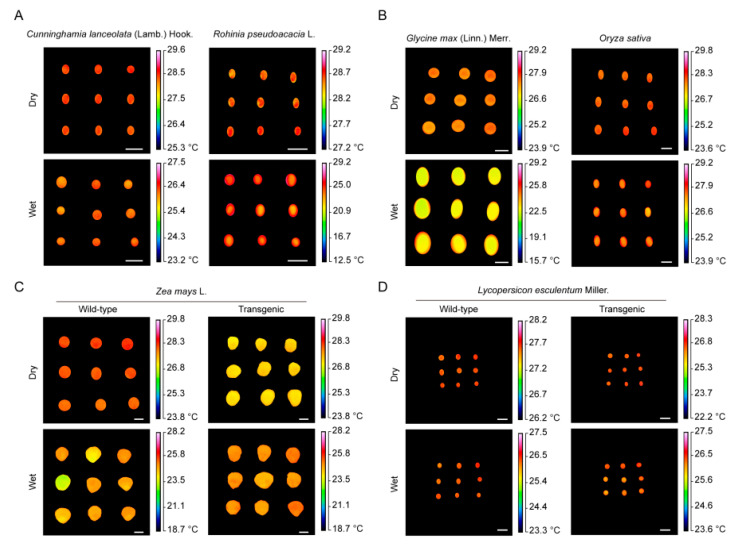
Thermal images of other seeds. Bar = 0.5 cm. (**A**). Thermal images of *C. lanceolate* seeds and *R. pseudoacacia* seeds (**B**). Thermal images of *G. max seeds* and *O. sativa* seeds (**C**). Thermal images for wild type and transgenic *Z. mays* seeds (**D**). Thermal images for wild type and transgenic *L. esculentum seeds*. The *Z. mays* and rice seeds showed differential temperatures between the wild type (WT) and mutants.

We also applied our infrared thermography system to other seeds that vary in seed size and different genotypes. Seed temperatures were measured for *C. lanceolata*, *R. pseudoacacia*, *G. max*, *O. sativa*, *Z. mays*, and *L. esculentum* (Figure 6). Interestingly, the *legumin 1* mutant line of *Z. mays* exhibited a notable difference in temperature compared with the wild type seeds (Figure 6).

## 4. Discussion

Seed deterioration is an irreversible biological process that inevitably occurs during seed storage. As seed vigor decreases, a series of changes takes place in the seed at the physiological, biochemical, and cellular levels [32,33,34]. Some damage has been observed to cell membranes during seed deterioration, resulting in increased permeability [35]. This damage greatly accelerates water absorption from the air in aging seeds, which theoretically leads to changes in heat absorption and reflection characteristics [20]. The water movement from the seed to the air is based on water vapour pressure deficit. In this case, seeds have less water content than that of air, so the reverse motion is expected especially if drying has been preceded. Respiration can be also a process accompanied by water production and heat release, which is affected by the aging process. Here, we provided experimental evidence of such changes in aging seeds using infrared thermal imaging.

Previous studies have documented that aging leads to a notable decrease in seed vigor, which decreases germinability [36]. In the present study, we observed considerable loss of germinability in *U. pumila* and *O. sativa* seeds after artificial aging treatments, due to a decrease in seed vigor, although they showed differential resistance to the aging treatments. The real-time thermal decay processes of *U. pumila* and *O. sativa* seeds were well characterized using the customized infrared thermal imaging system. Previously, thermal imaging technology has been used to capture temperature distributions in seeds of lettuce, *Pisum sativum*, *Triticum aestivum*, and *Brassica napus* [20,31]. Here, we also observed temperature distributions in seeds of *C. lanceolata*, *R. pseudoacacia*, *G. max*, *Z. mays*, and *L. esculentum*. Given that these seeds vary considerably in size, infrared thermal imaging can be applied to a broad spectrum of seeds.

Recent efforts have demonstrated tremendous value of infrared signals in non-invasive estimation of seed vigor [20,31,37]. Esteve et al. (2012) discriminated heat-damaged corn (*Z. mays*) kernels from viable ones using infrared spectroscopy [37]. Kranner et al. (2010) profiled heat emission from viable and artificially aged seeds of *Pisum sativum* in the processes of water imbibition and germination [20]. Although these methods discriminated aged from viable seeds, they rely on pre-acquired knowledge about chemical compounds in seeds to develop calibration models [37,38]. Moreover, when the seeds are imbibed and germinated to explore their infrared thermographs, there might be some loss in seed reserve, due to largely increased respiration [20]. Recently, mid-infrared lifetime imaging was used to characterize thermal decay processes of seeds [31,39]. It was found that the thermal decay lifetime parameter *d* exhibited the most significant variation between viable and aged seeds, which allows re-construction of distinct thermal decay delay-time images for viable and aged seeds [31,40]. In the current study, we adopted far infrared thermography to characterize real-time thermal decay of seeds with various viability. Here, we found that the thermal decay amplitude parameter *c* was the most closely related to seed vigor in *U. pumila*, *O. sativa*. We further established a close relationship between thermal decay amplitude and seed vigor, which permits quantitative predictions of seed vigor. The regression models showed excellent fit in *U. pumila* and *O. sativa*, which suggests potential value of this quantitative method to estimate seed vigor in various species.

Seed screening using infrared thermal imaging can be further developed to enhance screening accuracy and efficiency. Compared with traditional methods, including evaluations of germinability, conductivity, and TTC or bromothymol blue reagent staining, infrared thermography technology offers the advantages of no contact, higher sensitivity, and higher efficiency [20,37,40]. The present study documented a strong correlation between the thermal decay parameter *c* and seed vigor. Future studies should explore what and how questions of the parameter *c*. What structures or chemicals in seeds determine the *c*? How is the *c* related to seed vigor? In addition, future use of thermal imaging in seed vigor tests will be broadened when additional thermal markers are found.

The current study also demonstrated that our method can reliably estimate seed vigor regardless of seed water content. Although water absorbs infrared light at some wavelengths [41], which could interfere with measurements of infrared emission from seeds, we found that the thermal decay parameter *c* remained constant in dry and imbibed seeds. These results strongly support the use of parameter *c* as an indicator of seed vigor. Previous studies have reported that infrared thermal imaging can be used to isolate *Arabidopsis* mutant seedlings that are defective in stomatal regulation [30]. Similarly, we found that transgenic lines of *Z. mays* also showed distinct seed temperatures in our thermal imaging studies, suggesting that thermal imaging can also be used to isolate mutants at the seed stage.

## 5. Conclusions

In summary, the current study developed a new method for non-destructive evaluation of seed vigor. In particular, the close correlation observed between the thermal decay lifetime and seed vigor will facilitate quantitative prediction of seed vigor, thus providing time-saving and non-destructive seed vigor management in future silvicultural researches, as well as in agricultural researches.

## Figures and Tables

**Table 1:** The correlation analysis between thermal decay parameter and seed vigor of *U. pumila*.

Seed Vigor of *U. pumila*	*a*	*b*	*c*	*d*
Correlation coefficients	0.77	−0.83	−1.00	0.71
*p* values	0.07	0.04	0.00	0.11

*a*, *b*, *c*, and *d* are the thermal decay parameters.

**Table 2:** The correlation analysis between thermal decay parameter and seed vigor of *O. sativa.*

Seed Vigor of *O. sativa*	*a*	*b*	*c*	*d*
Correlation coefficients	0.77	−0.20	−0.83	−0.37
*p* values	0.07	0.70	0.04	0.47

*a*, *b*, *c*, and *d* are the thermal decay parameters.

**Table 3:** The regression models of seed vigor and the thermal decay parameter *c* for *U. pumlia* and *O. sativa.*

Regression Equation	*Ulmus pumila*	*Oryza sativa*
Polynomial *	y = 1292c^2^ − 1203c + 276 *R*^2^ = 0.94	y = −624c^2^ + 10c + 220 *R*^2^ = 0.77
Power	y = 9.67c^−1.29^ *R*^2^ = 0.84	y = 0.24c^−7.79^ *R*^2^ = 0.57
Exponential	y = 140.70e^−3.35c^ *R*^2^ = 0.76	y = 111519e^−15.25c^ *R*^2^ = 0.58
Linear	y = −152.71c + 104.86 *R*^2^ = 0.62	y = −633.87c + 384.52 *R*^2^ = 0.77
Logarithmic	y = −61.26ln(c) − 20.30 *R*^2^ = 0.75	y = −324.50ln(c) − 158.62 *R*^2^ = 0.77

* The polynomial function model gave the best fits for seeds of both *U. pumila* and *O. sativa* and *c* is the thermal decay parameter.

## Supplementary Materials

The following are available online at https://www.mdpi.com/2223-7747/9/6/768/s1. TTC means triphenyl tetrazolium chloride.
