# Supplementary material for: A Non-Invasive Analysis of Seed Vigor by Infrared Thermography
Source: Plants (Basel). 2020 Jun 19;9(6):768. doi: 10.3390/plants9060768 (PMC7356526; doi:10.3390/plants9060768)
Supplement: Supplementary file 1 [file plants-09-00768-s001.pdf]

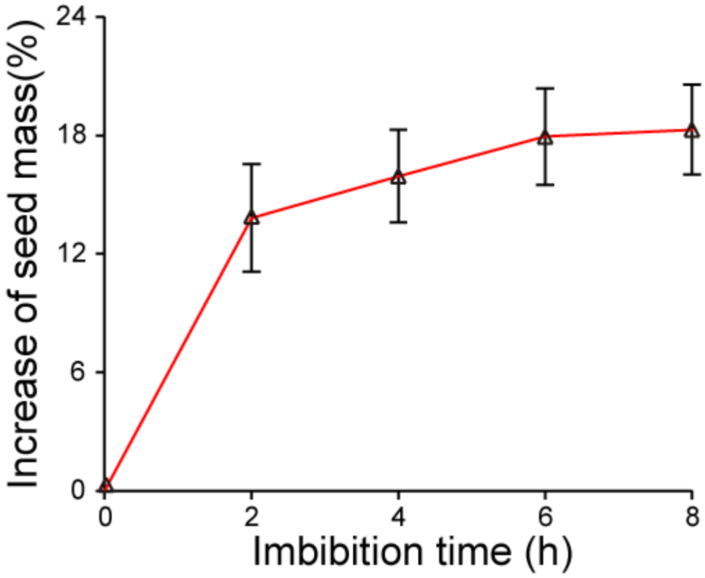

Figure S1. The imbibition curve of *U. pumila* seeds. Data are means ± SE.

Table S1. The ranges and coefficient of variation (CV) of T_{max} and T_{min} for *Ulmus pumila* seeds at different aging status.

Ageing period	T_{max}		T_{min}	
	**Range**	**CV**	**Range**	**CV**
0 h	(33.67, 34.15)	0.45%	(32.7, 33.16)	0.44%
24 h	(30.36, 34.08)	5.39%	(29.32, 33.11)	5.63%
48 h	(28.7, 33.72)	6.31%	(28.02, 32.81)	6.24%
72 h	(28.6, 33.74)	6.16%	(28.06, 32.75)	5.98%
96 h	(28.63, 33.68)	6.34%	(27.97, 32.67)	6.64%
120 h	(28.41, 30.89)	2.94%	(27.13, 30.08)	3.06%

T_{max} is the maximum temperature, and T_{min} is the minimum temperature of a seed during the thermal decay process. n = 12 for each measurement.
